# Supplementary material for: Round-trip migration and energy budget of a breeding female humpback whale in the Northeast Atlantic
Source: PLoS One. 2022 May 27;17(5):e0268355. doi: 10.1371/journal.pone.0268355 (PMC9140263; doi:10.1371/journal.pone.0268355)
Supplement: S1 File — (DOCX) [file pone.0268355.s001.docx]

**S1 Supporting information**

**S1 Table Sighting history based on photographic matching of individual fluke patterns**.

| **Season** | **Area** | **First sighting** | **Last sighting** | **Credit** |
| --- | --- | --- | --- | --- |
| 2013/14 | Kaldfjorden | 4-Dec | 5-Dec | Lars Kleivane, Fredrik Broms |
| 2014/15 | Skulsfjorden, Kaldfjorden | 20-Nov | 14-Dec | Fredrik Broms |
| 2019/20 | Kvænangen | 8-Jan- | 8-Jan | Audun Rikardsen |
| 2020/21 | Kvænangen | 2-Nov | 6-Jan | Audun Rikardsen |

**S2 Table Theoretical energetic cost at lower swim speeds during northward migration**

Our method differed slightly to that reported by (7, 39) since we integrate the cost of transport over time for each timestep. Therefore, we calculated the cost of the migration at slower average swim speeds of 0.9 ms^-1^(reported by (7) for mother calf pairs) and 1.1 ms^-1^ (optimal swim speed of mother calf pair reported by (39)) for the northward migration. To do so, we needed to obtain a vector of speeds with a similar distribution of speed values, but with a slower mean speed. We subtracted 0.37 (0.2 respectively) from each value of our original speed vector, then used the absolute values of this new resulting vector which now had a mean speed of 0.92 ms^-1^ (1.13 ms^-1^ respectively) and recalculated the cost. We also removed the last values to calculate the cost after 62 days, i.e., if the whale had stopped migrating after the mean migration duration reported by (7).

| Parameter | a)  Slower speed | B) Optimal SPeed | C) Shorter duration, slow speed |
| --- | --- | --- | --- |
| Duration (days) | 71 | 71 | 62 |
| Mean swim speed (ms^-1^) | 0.92 | 1.13 | 0.92 |
| E_COT_ (MJ) | 5 276 | 7 687 | 3 667 |
| metabolic maintenance (MJ) | 47 738 | 47 738 | 41 395 |
| E_TOTAL_ (MJ) | 53 014 | 55 425 | 45 062 |
|  |  |  |  |


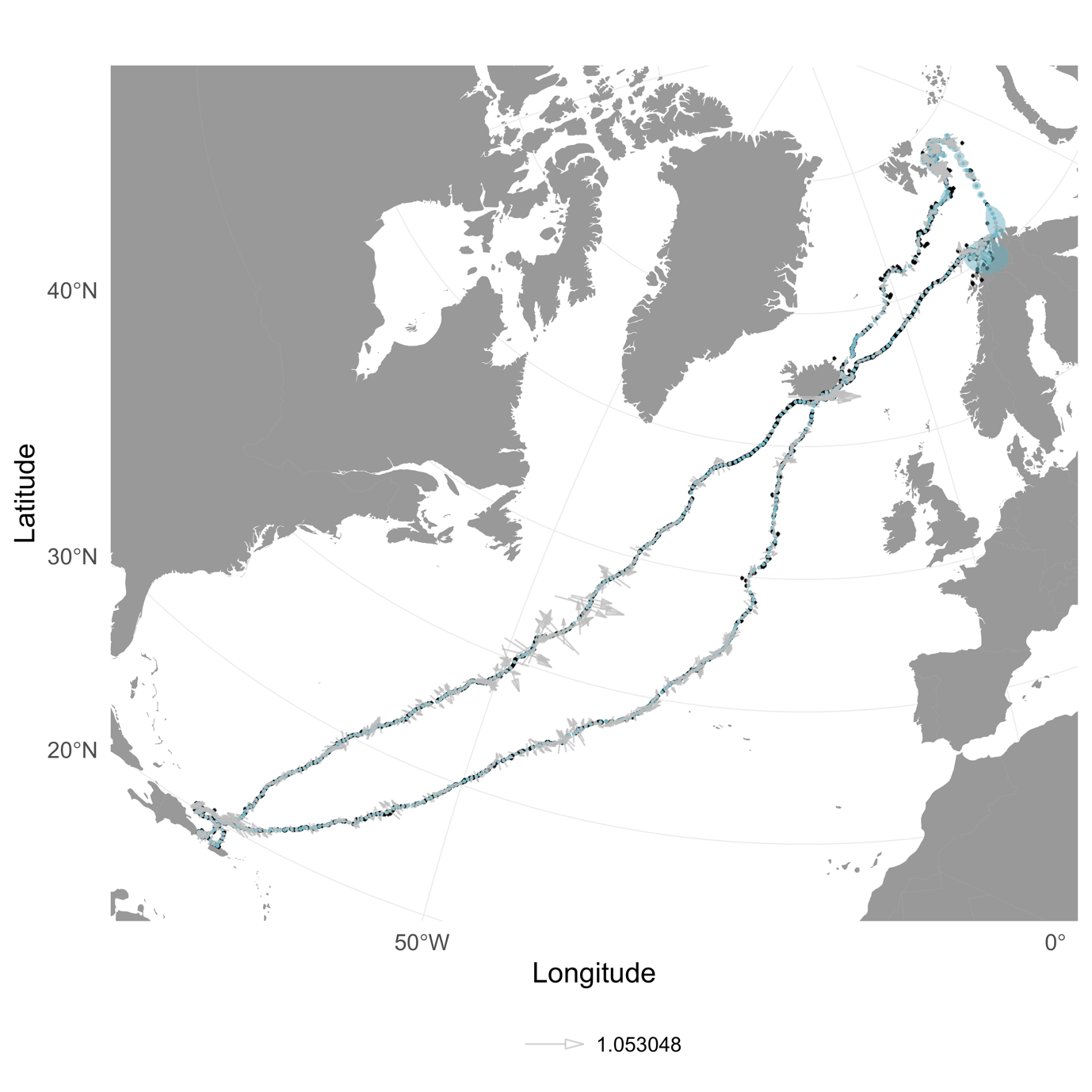


**S1 Fig** **Ocean currents and uncertainty of locations along track** The figure shows the reconstructed path in blue (sampled 6 hourly), including model-derived uncertainties (transparent blue) which increase towards the end of the tracking period, and observed Argos locations in black. Grey arrows indicate the current vector (direction and magnitude, (2)).

**S2 Fig Influence of ocean currents on whale speed** The influence of ocean currents in our analysis is presented as the difference between speed over ground and speed through water. Dates at which ocean current data was not available are indicated in red.
