# Supplementary material for: Round-trip migration and energy budget of a breeding female humpback whale in the Northeast Atlantic
Source: PLoS One. 2022 May 27;17(5):e0268355. doi: 10.1371/journal.pone.0268355 (PMC9140263; doi:10.1371/journal.pone.0268355)
Supplement: Attachment — Submitted filename: Response_to_Reviewers.pdf [file pone.0268355.s004.pdf]

Dear Dr. Paiva, dear editors,

We would like to thank you for considering our manuscript “Round-trip migration and energy budget of a breeding female humpback whale in the Northeast Atlantic” for publication in PlosOne. This manuscript has not been submitted elsewhere, and all coauthors have approved the submission of this revised version.

Our sincere thanks to you and the three Reviewers for a very thorough and constructive review of our submitted manuscript, which has greatly improved the work. We have considered each comment carefully and adapted the manuscript according to each Reviewer’s remarks and requests, as outlined below.

In particular, we slightly adapted the method of calculating the energetic cost of transport, since the Reviewers have correctly pointed out that there were inconsistencies in the units and integration of power over time. Since this makes direct comparison to a previously published study difficult, we have elected to include an additional results table (Table S2) in the supplemental materials to assess the energetic cost at theoretical slower mean speeds and shorter duration (as had been reported by other publications), and remove direct comparison statements.

We also slightly re-ordered the discussion after adding what had been requested by the Reviewers. To improve flow and readability we have also removed some repetitions, and shortened the abstract, maintaining the message of the text.

Further, we suggest to make the dataset available as follows upon acceptance:

- A minimal reproducible dataset is available in S2 Supporting information (csv format, incl. daily locations and all data underlying the reported values, i.e., distance between all locations, speed, movement persistence).
- To facilitate viewing of the data and improve findability, the data is available on Movebank.org, where we will link to this publication (ID 1064984327).

The R code used for the analysis is available on Gitlab:
https://gitlab.com/liserbeth/full-circle-humpback/-/blob/master/Full_circle_submit.Rmd

We hope that the Reviewers and editors find the work improved and look forward to any further comments.

Sincerely,

Lisa Elena Kettemer, Ph.D. candidate

on behalf of the authors.

Reviewer 1 comments

1. 28: “longest [mammal] migration distance recorded”.

We inserted the missing word.

2. For capital/income breeding strategies, I would suggest citing one or more of the following foundational papers. Drent and Daan (1980) https://doi.org/10.5253/arde.v68.p225 ; Jönsson (1997) https://doi.org/10.2307/3545800; Stephens et al. (2009) https://doi.org/10.1890/08-1369.1

We have included references to Jönsson (1997) and Stephens et al. (2009).

3. 102: What does RIB stand for? I assume rigid-hull inflatable boat, but it should be defined before first use.

We have replaced the acronym with the full name (rigid-hull inflatable boat) since it is not used again in the text.

4. 103: Is ARTS an acronym or a brand?

We have changed this to “Aerial Rocket Tag System (LKARTS)”, as it is both an acronym (ARTS, removed) and a brand (LKARTS).

5. 111: Please specify the version of R.

Implemented.

6. 113: Is there a reason you used a projection? In other words, why not use geodesic or great circle distances instead? I think you’d get more accurate step lengths that way.

We project data before modeling the most likely path, since the fit.ssm() function in the foieGras package requires data to be projected. The default argument uses a standard Mercator projection if none is specified. For our data spanning from the Barents Sea through the Caribbean this would lead to distortion between the different sections of the track. We used an azimuthal equidistant projection centered on a location at the middle of the track. This projection should best conserve distances across the entire range.

To calculate displacement between the predicted locations along the modeled path, we previously used the adehabitatLT package, which gives euclidean distances between unprojected coordinates. We chose to use the geodist package as suggested. The difference in speed that resulted from this change in methods resulted in only negligible deviations from previously reported speeds and did not influence our results. Numbers have been updated throughout the text.

7. 116: For the trip package, the authors request you also cite Sumner's dissertation (details available at https://cran.r-project.org/web/packages/trip/citation.html). Also, please specify the package version (presumably 1.8.5). I appreciate that you cited Freitas et al. (2008) for the sda function. For the sake of clarity, I would suggest stylizing function names as `function()` (i.e., fixed-width font like Courier New and followed by parentheses). In my opinion that improves readability.
8. 119: For the foieGras package, the authors request you also cite Jonsen et al. (2020) Movement Ecology and Jonsen et al. (2019) Ecology (details at https://cran.r-project.org/web/packages/foieGras/citation.html). Also, for Jonsen and Patterson (2020), please include the DOI. It's a Zenodo archive, so without the DOI your readers won't be able to find it. Please specify the package version (0.7-6, I assume). For consistency, since you specified using trip::sda(), I think you should also specify which function you used from foieGras (presumably foieGras::fit_ssm()?)
9. 130: Specify the version of adehabitatLT used. This is especially important because the paper you cited (Calenge (2006) Ecological Modelling) predates the author splitting adehabitat into multiple packages. Here again, I would suggest stylizing the function name as lavielle(). Calenge also recommends citing Lavielle (1999) https://doi.org/10.1016/S0304-4149(99)00023-X.
10. 139: What version of marmap did you use? The latest version (1.0.6) doesn't have a function called lc.cost(). There is a lc.dist() function – maybe that one?

We thank the Reviewer for the above suggestions and have implemented them throughout the text. We clarified the versions and functions used.

11. BMR estimates are a critical component of this analysis and there is a great deal of uncertainty around the metabolic rates of very large animals (i.e., the Kleiber curve was fit to data collected from species orders of magnitude smaller than a baleen whale). I am curious whether the results presented here are robust to assumptions about metabolic rate scaling. For example, White and Seymour (2003) argued the 3/4 power law is an artifact of large terrestrial mammals' digestive physiology and the "true" exponent is actually 2/3 (https://doi.org/10.1073/pnas.0436428100). Their allometric equation, when extrapolated to 30 000 kg, would yield much lower BMR. Conversely, Kolokotrones et al. (2010) found evidence for metabolic scaling curvature in log-log space (thttps://doi.org/10.1038/nature08920). Their quadratic allometric equation predicts elevated BMR in a humpback relative to Kleiber. I think the arguments in this paper would be strengthened by repeating the analysis with BMR estimates predicted by these equations. Does the finding that "pregnant females migrate faster than the energetic optimum" hold if BMR is higher than the Kleiber prediction?

We agree with the Reviewer that a range of the parameters used in the bioenergetic model warrant an updated assessment (e.g., the drag coefficient). We have taken this into consideration, however one of the main aims of this paper was to compare this migration to other humpback whale migrations, given the specific characteristics of the migration from Norway (duration, seasonality, distance) and to assess how the costs compare to migrations in other parts of the world. We think that a dedicated sensitivity analysis is warranted for bioenergetic models assessing the energetic cost of humpback whale migrations in general,

however we do feel that is outside the scope of this current manuscript. However, we have provided further discussion on this issue in the discussion section (l.390-407). Our conclusion is that while we can determine that the cost of this migration was higher than those of humpback whale migrations covering shorter distances, or similar distances at slower speeds, the magnitude of this difference is subject to change given our limited knowledge of the parameters. The text now reads as follows:

"Obtaining reliable measurements of biomechanical parameters and travel speeds is challenging for large marine vertebrates, so bioenergetic models such as the one presented here, rely on a range of assumptions and approximations (42,80). While parameter values in the model are averages of estimates, true values are likely variable between individuals and over time (due to differences in mass, condition, surface area, gaits, appendage morphometrics, behavioral patterns). For example, we use a fixed value for the drag coefficient (C_d) which has been estimated by previous studies. Substantial uncertainty exists around this value for most species, including humpback whales ((6) but see (78) and (81)), and it also varies with many of the same factors mentioned above. Similarly, BMR cannot be directly measured for large free-ranging marine animals, and there is disagreement on the relationship between size and metabolic cost for large animals (82,83), so we rely on estimates based on allometry. Since both C_d and BMR are central parameters in this model, our estimates are only rough indicators. Importantly, changing the values for BMR and C_d in the bioenergetic model may lead to a shift in the relative importance of maintenance costs vs. transport costs, i.e., the importance for a whale to minimize metabolic costs (by decreasing the duration of migration) vs. minimizing transport costs incurred from movement (by decreasing speed). By using the same parameters as previous studies we can ascertain that the relative cost of this migration is larger than that reported previously (7), but the magnitude of the increase cannot be determined with certainty."

12. 155-156: You define propulsive efficiency as "how efficiently muscle work is converted to forward motion" but I think that conflates propulsive and aerobic efficiency. I believe it is more accurate to say "how efficiently mechanical work is converted to forward motion".

We agree with the Reviewer and now use the suggested definition.

13. Out of curiosity, how much was the contribution from ocean currents to displacement? Also, did you account for declining current speeds with depth? My (admittedly untested) assumption is current contributions would be negligible, but if your calculations indicate otherwise I would like to see that. Could you add a figure to the supplemental materials plotting corrected vs uncorrected speeds?

We have added a supplemental figure (Fig. S2) to show that the influence of ocean currents on whale speed was only small (the median of the absolute difference between speed over ground and speed through water was 0.07 m/s). We used the surface currents (the upper layer in the dataset comprised 0-5m depth) and did not account for the effect of dive depth (no depth information was collected from this tag). We have added a statement in the manuscript, which now reads (l.414-419):

“Furthermore, animals likely travel at a depth where additional wave drag produced at the air/water interface is minimized (e.g., ~12 m for large baleen whales) (82). Current speed at increasing depth may differ to those at the surface. As the tag used in this study did not collect dive information, we assume that the whale swims at an optimized depth most of the time, avoiding additional wave effects at the surface, and that the ocean current effects experienced can be approximated by surface currents (0-5 meters depth).”

14. 170: Was there a particular function you used for estimating SOG?
15. 173-174: windR is in a living repository (as opposed to archived on CRAN or Zenodo) and there are no tagged releases, so please add “date accessed” information to the reference. Also, did you use a particular function? I would guess windSupport().

We clarified this on l.194:

“We followed (44) using windSupport () in the package windR (41, accessed 15.02.2021).“

16. 196-198: I take it these distances were calculated using the projected coordinates, correct? How different are they if you use geodesic distances? At these spatial scales I think it’s possible the errors could add up to a substantial amount.

We agree that at these scales geodesic distances should be used to calculate the displacement distances. We therefore now use the geodist() function in the geodist() package, specifying method=”geodesic” (see also our response to Reviewer comment 5).

17. 226-227: I think these units are wrong. Watts are a measure of power (energy per unit time) and I believe you summed power over time, so this should be in Joules.

We agree with the points raised by the Reviewer, and have updated the method, as it had not properly included the integration over time. We also include a link to the code, if the Reviewer would like to see the exact approach used. The updated method is described as follows (l. 173):

“Equation (2) gives the instantaneous power required to overcome drag (in Watt, or Joule s^{-1}) at a given speed. Our data consists of interpolated positions at 6-hour intervals, providing swim speed estimates for each of these intervals. To convert this instantaneous power (Eq 2) to the energy expenditure required to swim at this speed for the duration of each interval, we therefore multiply it by the timestep duration (i.e., 6 hours). To obtain an estimate of the daily cost of transport, we then sum every set of four 6-hour estimates. ”

18. 279-293: Your results about the timing of calving are fascinating! Very excited to see this recorded with tag data.

Thank you!

19. This is largely why I'm curious about how much ocean currents contributed to swimming speeds. How much could the other studies be off by?

We have added a supplemental figure to show the influence of ocean currents on whale speed was only small, and therefore the difference in speed we observed was unlikely due to this difference in method. We now provide an additional figure in the supplemental materials (S2 Fig.) and mention this aspect in the manuscript, l. 348:

"None of these studies accounted for ocean currents, but speed was overall only slightly influenced by currents (mean absolute difference between speed through water and speed over ground 0.07 ms^{-1}, S2 Fig in S1 Supporting information). "

20. I find this particularly interesting, as it means time, not energy, is the important currency. To me, this suggests rorqual whales are such efficient foragers that they are essentially food unconstrained during the feeding season. What does it matter if you burn more calories during migration if you're a feeding powerhouse for a few months of the year? Relative to their body size, I think rorqual whales may actually have very fast-paced life histories. Perhaps it's out of the scope of the present manuscript, but I'm curious what the authors think their results say about pace-of-life at extreme body sizes.

We would argue that the high seasonal herring abundance in this particular fjord area may be enough to offset the additional 500 km caused by the detour, and the faster speed during the migration. Assessing whether the observed behavior is generalizable across the population will shed more light on this. We also agree with the Reviewer that some of the parameters in the energetic model should be re-assessed using a larger dataset, which will provide further insights into the importance of time vs. energy minimizing strategies during humpback whale migration. Investigating these questions at the extreme end of the recorded migratory distances for mammals will help us better understand the pace-of-life of large baleen whales and we look forward to further discussions on this topic.

21. Table 1: You cite references 66 and 67, but your bibliography only goes up to 63. Also, I don't see 64 or 65 cited anywhere.

The errors in the reference list and Table 1 have been corrected.

22. Regarding drag, a recent study (Gough et al., 2021) using tag data derived a drag coefficient five times greater than the value used in the model (https://doi.org/10.1242/jeb.237586). Granted, that analysis looked at a finer temporal scale than migration, but it does raise questions about the model's parameterization. Based on equation 2, ECOT is directly proportional to CD. Do the authors' results and interpretations change if ECOT is 5x greater?

When using the updated estimate for the drag coefficient, the cost of transport increases, as the Reviewer states. We consider the uncertainty around the energetic cost estimate large, and now state on l.390-407 that our main conclusion is independent of these uncertainties (the cost is

higher for this migration than for others). Importantly, the relative cost of maintenance cost and locomotion cost may change as the parameters change, but we think that it will be more useful to focus on assessing these effects in future studies with a larger number of tracks. Please also see our response to Reviewer comment 11.

23. Table 2: The units in this table are very confusing to me. E KM-1 should be in units of J km-1 maybe? It's not W, which is energy per unit time (not distance). Similarly, I can't understand E DAY-1 (W), since W is J s-1, not J day-1. I believe ECOT, BMR, and ETOTAL should be in J, not W, since you integrated over time (if I understand correctly). I think BMR should be "Total basal metabolic expenditure" or something like that, since after integrating you're no longer talking about a rate.

We have updated the method and now report results in Joule and Joules/day respectively (see response to Reviewer comment 11). We elected to remove the cost per unit distance as it was misleading, since the cost for the entire migration includes time spent on the breeding grounds (during which no substantial distance was covered). We included a link to the code, if the Reviewer would like to see the exact approach used.

24. Figure 1: I really like these maps. Just a couple comments. 1) I would change the legend title of the inset from "g" to "" to better match the text. 2) I think the inset legend would be cleaner if you expanded the limits to include 0 and 1. 3) In the main map, you overlay red boxes on green points. It's unclear what the red boxes represent. I assume the West Indies red box delineates the inset, but I'm unsure what the four northern boxes are. Also, people with color blindness will have a hard time interpreting red on green, so please choose a colorblind friendly combination of colors. Lastly, you cite (68) but the bibliography only goes up to 63.

Thank you for pointing out that the color choice was not colorblind friendly. We implemented the suggested changes and the bibliography has been updated for the figure files. The boxes indicated the locations at which breakpoints were detected, but since this is indicated by the color fill, we have removed this to avoid confusion and crowding of the map.

25. Figure 3: Great figure. My only request is you make the movement persistence gradient in this figure match the gradient in the figure 1 inset, for consistency.

Thank you. We have implemented the suggestion.

26. Figure 4: This is a helpful figure, but I'm unclear how you calculated the first and third quartiles. If the dotted lines represent Q1 and Q3 for each segment, then shouldn't there be the same number of points below the Q1 line and above the Q3 lines? For example, the second segment appears to have 9 points below Q1 and 19 above Q3. The next segment has only two points above Q3 and 13 below Q1. How did you come up with those quartiles? Also, and this is really nitpicky, the interquartile range is (technically speaking) the difference between Q3 and Q1 (i.e., it's a single number). I think it would be more accurate to say "dotted lines represent the first and third quartiles".

We have corrected the calculations for the quartiles, using the stats::quantile() function and using the 2nd and 4th entry of the resulting vector (1st and 3rd quartiles) for plotting. The figure legend now reads:

"…dotted lines represent the first and third quartiles".

—---------

Reviewer 2 comments

1. Line 71-72: "They may therefore face higher energetic constraints compared to whales in the western Atlantic" – you should provide more context for this comparison by mentioning the breeding and feeding grounds between which the whales in the western Atlantic migrate, as most readers might not know what they are and can't form a mental image of how and why the NE Atlantic humpbacks would face a higher energetic constraint compared to the W Atlantic whales.

We thank the Reviewer for pointing out that this may be unclear. We agree and have added a clarification. The text now reads (l.66):

"The distance between the Barents Sea and the West Indies represents the longest migration route of any humpback whale population (a great circle distance of ~ 9 000 km vs. 8 461 documented by (9)). Northwest Atlantic humpback whales also migrate to the West Indies from Newfoundland-Labrador, the Gulf of St. Lawrence, or the Gulf of Maine (15,17–19), a distance up to 5 000 km. As a result of this long migration distance, NEA humpback whales may face high energetic constraints compared to whales migrating elsewhere. "

2. Line 76: "Norwegian spring spawning (NSS) herring" – NSS acronym not necessary as not mentioned again in the manuscript

We agree and have removed the acronym.

3. Lines 225-227: "We estimated the total cost of the round-trip migration (from coastal Norway to the Barents Sea) to be 2 681 937 Watts (W), where 51% (1 365 516 W) constitutes cost of transport and the rest represents maintenance metabolism (1 316 420 W, Table 2)" -- I think you should briefly mention (probably in the Discussion) the uncertainty around this exact W value provided, due to the uncertainty of the various parameters (e.g., for some of the parameters there is no exact value for humpback whales, plus the value of mass used in the model may not have been the exact size of the individual whale etc. etc.).

We have included a paragraph on l. 390-407, detailing the uncertainties in the parameter space and how these may impact our results. This part of the discussion now reads:

"Obtaining reliable measurements of biomechanical parameters and travel speeds is challenging for large marine vertebrates, so bioenergetic models such as the one presented

here, rely on a range of assumptions and approximations (42,80). While parameter values in the model are averages of estimates, true values are likely variable between individuals and over time (due to differences in mass, condition, surface area, gaits, appendage morphometrics, behavioral patterns). For example, we use a fixed value for the drag coefficient (C_d) which has been estimated by previous studies. Substantial uncertainty exists around this value for most species, including humpback whales ((6) but see (78) and (81)), and it also varies with many of the same factors mentioned above. Similarly, BMR cannot be directly measured for large free-ranging marine animals, and there is disagreement on the relationship between size and metabolic cost for large animals (82,83), so we rely on estimates based on allometry. Since both C_d and BMR are central parameters in this model, our estimates are only rough indicators. Importantly, changing the values for BMR and C_d in the bioenergetic model may lead to a shift in the relative importance of maintenance costs vs. transport costs, i.e., the importance for a whale to minimize metabolic costs (by decreasing the duration of migration) vs. minimizing transport costs incurred from movement (by decreasing speed). By using the same parameters as previous studies we can ascertain that the relative cost of this migration is larger than that reported previously (7), but the magnitude of the increase cannot be determined with certainty."

4. Also, because we know for sure that this individual was gestating and then lactating (lactation being hugely energetically demanding), I think it should be mentioned somewhere how the exact W value will be an underestimate of the TRUE energy amount (W) that the animal used (although 51% of this estimate without lactation was transport costs, 51% of the TRUE round-trip cost probably wasn't travel….). And a follow up to that point, did you consider adding lactation cost to the model (unsure if that exists for humpback whales…) - how would that have affected the calculations and results? It could be worth addressing why lactation cost (and gestation cost) was not added into the calculations, as most people will probably ask about that. But I think the discussions and comparisons around the cost of transport are fine as the (only) key parameter there is the speed of travel.

Given the uncertainties in the parameter space of the bioenergetic model (now discussed in more detail in the manuscript), we decided to not estimate the cost of gestation and lactation. A general model is provided by Braithwaite et al. (2020). We briefly mention in the discussion how the cost of lactation may be influenced by the behavior observed in this study. Since there is no reliable way of quantifying this we have elected to omit presenting an estimate. See l.371:

"Reduced resting time and faster swim speeds can lead to a loss of milk and increased energetic demands of the calf, increasing the energetic cost to the mother beyond that solely caused by higher costs of transport at higher swim speed (6). Because we cannot reliably quantify this effect (but see (6)), we did not include the cost of lactation and gestation. Therefore, the total energy expended by the mother during migration will be higher than reported."

5. Lines 239-241: "Earlier studies on humpback whales in other regions have only mapped their migrations one way" - it could be worth mentioning here that this isn't necessarily due to lack of trying but due to lack of data as the tags haven't lasted long enough to capture full migrations

(leading to an optional discussion point: developments in the tags, perhaps the newer tags such as used here are better in some ways?).

We have included a brief statement on this on l. 282, but have chosen not to discuss in detail the technical aspects of tag deployment durations, as we believe it would require a more detailed analysis than we can include here:

"While earlier studies have provided key insights into humpback whale movement and migrations in many other regions, these studies have only mapped migrations one way, due to the limited longevity of tag deployments."

6. "most individuals likely travel directly from the main foraging grounds to breeding grounds" – given that you calculated the most direct route and showed that the tagged individual did not take the straightest possible route when migrating, I think there is a great chance to add some broader (brief) discussion on 'why don't animals just take THE shortest route possible', and in the case of this whale what might be the reasons for not taking the most direct/shortest route (e.g. is it because they learn the route from their mother…?)

In the interest of keeping the manuscript relatively short, we have elected to not discuss this question in the manuscript. However, we agree that this is an interesting aspect of whale migrations. Notably, the difference between the shortest possible path and the actual trajectory on the northward migration (during which no stop-over detour was taken) was only ca. 500 km, not a very high difference given the extremely long overall migration. So, we think humpback whales are fairly good at finding an efficient route, and other studies have speculated on different mechanisms that allow them to do so, and have attempted to tease these apart by analyzing tracking data (see e.g., Horton et al. 2011 and 2017[1]).

7. Lines 266-268: "The departure timing from Norway on February 7th is slightly later than that of other whales feeding in coastal Norway, as they generally depart between December and late January (16)." – I think you should expand here about this 'late departure' and why that might be – the hierarchical migratory timing of humpbacks. You already mention this a bit in the introduction (lines 85-86: "While pregnant humpback whales commonly maximize the time spent on feeding grounds (17–19)").

We expanded our discussion to this regard and l.308 now reads:

"The whale left Norway on February 7th, slightly later than other whales , as most leave between December and late January (23). In other regions, pregnant females also remain up to two months longer on the feeding grounds compared to other groups (24–26), presumably to cover the substantial additional cost of pregnancy and lactation (58,59). Females can maximize

---

[1] Horton et al. (2017) Frontiers | Multi-Decadal Humpback Whale Migratory Route Fidelity Despite Oceanographic and Geomagnetic Change | Marine Science
Horton et al. (2011) Straight as an arrow: humpback whales swim constant course tracks during long-distance migration | Biology Letters

their calf's chances of survival by providing sufficient energy and resources both during pregnancy and subsequent lactation (60,61). This strategy may increase reproductive success of the mother if it is successfully employed across years (24,58,62)."

8. Lines 289-290: 'humpback whale calving events in the Southern Ocean have been reported as far south as 15 -20°S, outside of the main breeding grounds" – when you say "calving events in the SO outside of the main breeding grounds", I wonder what you mean exactly… I think this sentence needs some edits as it doesn't sound quite right. The SO (starting at 60S) is the feeding ground for southern hemisphere humpbacks. Also, on the 'as far south as 15-20S' – in New Caledonia a key breeding site is the south lagoon which is around 22S, and therefore further south than the 'as far south as 15-20S'. The second half of this sentence on lines 291-292 "supporting the idea that births can occur over a large geographic area and within a 4- 6-month window" is really good, I just think that the first half of the sentence needs some adjusting and clarifying.

We thank the Reviewer for bringing this to our attention. We meant to refer to the southern hemisphere, rather than the Southern Ocean, but have chosen to adapt the sentence to focus rather on the documentation of neonate calves outside of breeding grounds. The sentence now reads:

"Newborn calves have been documented outside of the described main breeding grounds elsewhere (72,73) and historical whaling records from Norway include records of late-stage pregnancies in Norwegian waters during winter and spring (74), also indicating humpback whales from this region might give birth shortly after these observations were made, likely outside breeding grounds (75). "

9. Line 300: "none of these studies accounted for ocean currents." – Few thoughts around incorporating ocean currents in the speed calculations:
   1. I think you might want to briefly touch on any caveats with the data you used (e.g. missing data for some time periods (for what % of locations during active migration was current data not available? How might that affect the results?), ocean current data probably reflects surface waters and although humpbacks do hang around near the surface quite a lot they obviously wouldn't be affected the same way if they dive deeper?).

We thank the Reviewer for bringing these points up. Only 3 of missing ocean current data instances occurred during the migration, and we do not report speeds on the feeding grounds (where some data was missing). Therefore we have chosen to remove the sentence to avoid confusion. We have further provided a more detailed discussion on the caveats of using surface currents on l. 414:

"Furthermore, animals likely swim at a depth where additional wave drag produced at the air/water interface is minimized (e.g., ~12 m for large baleen whales) (86). Current speed at depth may differ to current speed at the surface. As the tag used in this study did not collect dive information, we assume that the whale swims at an optimized depth most of the time, avoiding additional wave effects at the surface, and that the ocean current effects experienced can be approximated by surface currents (0-5 meters depth)."

10. You make the point that other studies didn't account for ocean currents. What is the migration speed in your data if you don't account for ocean currents? It would be interesting to see what your results would be if you hadn't corrected for currents and would they then have been more similar to the previous studies?

We acknowledge that this was not sufficiently clear previously, and as also requested by Reviewer 1, we have included a figure in the supplemental materials (S2 Fig.), showing the difference between speed over ground and speed through water for each timestep. The median of the absolute difference between speed over ground and speed through water was 0.07 m/s. We have also added a brief statement on this on l. 348, which now reads:

"None of these studies accounted for ocean currents, but speed was overall only slightly influenced by currents (mean absolute difference between speed through water and speed over ground 0.07 ms^{-1}, S2 Fig in S1 Supporting information). "

11. Lines 319-320: "faster swim speeds can lead to a loss of milk and increased energetic demands of the calf," – I think you need to briefly mention how mothers and calves swim in echelon formation which allows the calf to slip stream and not use much energy (though on the flip side that changes the drag the mother experiences – you should acknowledge this as well as this change in drag was not included in the model).

We thank the Reviewer for bringing up this point and have added a statement in the discussion section on l.408, which now reads:

"Additional uncertainties exist regarding the energetic costs of mother-calf pair movement and how these scale with speed, since calves swim directly at their mothers' side, thereby changing the mothers' drag profile and their own (84). While optimal swim speeds seem to be largely independent of size across the range examined in a recent study (minke whales - blue whales (77)), calves have less muscular power and lower lung capacity than adults (85). Therefore, calf requirements and swim speed likely determine resting periods and overall migration speed."

12. Lines 322-338: This is a really interesting comparison! So the energetic cost of the northward migration for this one animal was twice the average cost reported for females migrating with calves from the Southern Ocean to Australia – but – the distance for this one animal was also twice as long, so technically that would mean the transport cost for a comparable distance (e.g. for every 1000km travelled) was actually the same. However, this one animal travelled a lot faster. I wonder what effect the inclusion of ocean currents in your model had here… Either way, it is an interesting chance to compare model results, and you do a nice job discussing the tradeoffs between travelling faster (and using more energy) vs having more time on the feeding grounds. I wonder if there has been or will be any work in the Barent Sea/Norway area to try to estimate the energy gained by humpback whales during the feeding season (as the annual energy budget will be a function of both energy used during the year and energy gained during the year).

We compared only the northward migration cost to the cost of the northward migration as presented in Riekkola et al. (2020)[2]. However, since we updated the method and values presented in Riekkola et al. (2020) were therefore not directly comparable. We now instead provide an additional calculation in the supplemental material. For this, we calculate the cost given a theoretical lower swim speed (as those reported by Riekkola et al. (2020) and Braithwaite et al (2015)[3]):

Table S.2

Our method differed slightly to that reported by (7, 38) since we integrate the cost of transport over time for each timestep. Therefore, we calculated the cost of the migration at slower average swim speeds of 0.9 ms^{-1}(reported by (7) for mother calf pairs) and 1.1 ms^{-1} (optimal swim speed reported by (37)) for the northward migration. To do so, we needed to obtain a vector of speeds with a similar distribution of speed values, but with a slower mean speed. We subtracted 0.37 (0.2 respectively) from each value of our original speed vector, then used the absolute values of this new resulting vector which now had a mean speed of 0.92 ms^{-1} (1.13 ms^{-1} respectively) and recalculated the cost (data in supplement 2). We also removed the last values to calculate the cost if the whale had stopped migrating after the mean migration duration reported by (7).

**Parameter**	**a) Mean Speed (7)**	**B) Optimal Speed (38)**	**C) Mean duration (7)**
**Duration** (days)	71	71	62
**Mean swim speed** (ms^{-1})	0.92	1.13	0.9
E_{COT} (MJ)	5 276	7 687	3 667
**Metabolic maintenance** (MJ)	47 738	47 738	41 395
E_{TOTAL} (MJ)	53 014	55 425	45 062

[2] Riekkola et al. (2020) Longer migration not necessarily the costliest strategy for migrating humpback whales | Aquatic conservation
[3] Braithwaite et al. (2015) Optimal migration energetics of humpback whales and the implications of disturbance | Conservation Physiology | Oxford Academic

As we show in another new supplemental figure (Fig. S.2), the effect of including ocean currents was small (the median of the absolute difference between speed over ground and speed through water was 0.07 m/s) and was therefore unlikely to be the reason for the higher speed and cost. In fact, only by increasing the speed was the animal able to “save on” metabolic costs by reducing the duration of the migration. We would like to point out again that the uncertainties of the parameter space mean that we may not be able to draw conclusions of the relative importance of metabolic costs to transport costs. Their relative importance determines the optimal strategy to conserve energy.

We have added a paragraph on the uncertainties in the discussion section on l. 390-407 (see also our response to Reviewer comment 3) and have discussed the influence of ocean currents in our response to Reviewer comment 10 for which we have updated the text on l.l. 348.

13. And now that I have read that section of the discussion, going back to the abstract lines 37-39: “The estimated energetic cost of this migration was substantially higher than the energetic costs of other humpback whale migrations, resulting from the long migration distance and fast migration speed (1.6 ms-1).” – you might want to be mindful of the wording, because if you are comparing the energetic costs of a full migratory cycle to only a half a migratory cycle then of course it will be higher. And based on the discussion section, if we just double the southern hemisphere half a migratory cycle cost then it actually ends up being about the same, i.e., no substantial difference between this migration cost and those in the southern hemisphere. Maybe just adjust the wording a bit so that you aren’t making claims that you don’t mean to/that aren’t backed up by your results.

We agree that this may be easily confusing, however we did in fact compare the cost of one-way migration (only northward) to the one-way migration reported in Riekkola et al. (2020). Please see our response to the previous comment (Reviewer comment 12). We hope our additions clarify the misunderstanding and believe we can demonstrate that the cost was larger (see also added calculations in supplemental material, Table S.2). We have removed the word “substantially” when comparing our results to the likely cost for a whale traveling at the mean reported speed and duration in Riekkola et al. (2020) throughout the text.

14. Figure 1: Because figures should be fully standalone from the text, I think you need to explain movement persistence in the legend a little bit for those readers who aren’t familiar with state-space models so that they don’t have to search for the information in the main text while looking at the figure. I would suggest the following: the ‘g’ in the scale should be mentioned in the legend e.g., by saying “… movement persistence (g)…” followed by something along the lines “ ..lighter blue colours indicating directional travel, and darker blue colours indicating meandering movement..”

We thank the Reviewer for these helpful suggestions, and have implemented them.

15. Also, it looks like in the insert the line is on top of the blue points, causing it to be a little hard to make out the difference between the lighter and darker shades of blue – I would suggest placing the line under the points so that the colour differences are clearer.

We have updated the color scale in the inset, making it now easier to identify the changes across the range of values.

16. Figure 2: maybe provide exact dates for the photos?

Implemented.

17. Table S.2: I don't think it would actually be of much relevance for your work, but some variability in migratory speed between the individual humpback whales in the southern hemisphere are provided in https://doi.org/10.1016/j.ecolind.2018.02.030 (although calculations were done at a 12 hour time step).

We thank the Reviewer for mentioning this reference, we included it on l. 357.
—---------

Reviewer 3 comments

Title: energy budget => energetic cost?

We have elected to maintain the word "budget", since we think the word "cost" would need to be followed by an explanation and make the title even longer.

L117 pre-filtered => filtered?

Implemented.

L174 current corrected swim speed => estimated swim speed through water?

Implemented.

Table 1. Lambda is <1, which means that animals incur less drag during active swimming than gliding. This seems to be a very unusual case (see, for example, Weihs 1973 J. Mar. Res.). I cannot find the cited reference (37). Please provide a regular article for the citation here. Also, papers with reference no. of >63 are not listed.

We have corrected the bibliography and all references are now correctly linked. This lambda value has been used in the referenced studies estimating the cost of migration in humpback whales, and the optimal migration speed, and since one of the main aims of this study was to assess the cost of the described migration in comparison to others we needed to use the same parameter values. However, we agree with the Reviewer that this parameter may need to be

reassessed for future studies. Indeed, some other parameters are being re-evaluated with the availability of new, high-resolution tagging (e.g. propulsive efficiency, drag coefficient see Gough et al. 2019, 2021[4]), and we now discuss this in more detail on l. 390-407:

"Obtaining reliable measurements of biomechanical parameters and travel speeds is challenging for large marine vertebrates, so bioenergetic models such as the one presented here, rely on a range of assumptions and approximations (42,80). While parameter values in the model are averages of estimates, true values are likely variable between individuals and over time (due to differences in mass, condition, surface area, gaits, appendage morphometrics, behavioral patterns). For example, we use a fixed value for the drag coefficient (C_d) which has been estimated by previous studies. Substantial uncertainty exists around this value for most species, including humpback whales ((6) but see (78) and (81)), and it also varies with many of the same factors mentioned above. Similarly, BMR cannot be directly measured for large free-ranging marine animals, and there is disagreement on the relationship between size and metabolic cost for large animals (82,83), so we rely on estimates based on allometry. Since both C_d and BMR are central parameters in this model, our estimates are only rough indicators. Importantly, changing the values for BMR and C_d in the bioenergetic model may lead to a shift in the relative importance of maintenance costs vs. transport costs, i.e., the importance for a whale to minimize metabolic costs (by decreasing the duration of migration) vs. minimizing transport costs incurred from movement (by decreasing speed). By using the same parameters as previous studies we can ascertain that the relative cost of this migration is larger than that reported previously (7), but the magnitude of the increase cannot be determined with certainty."

Hind and Gurney (1997)[5] (where this lambda value is derived from) also discuss that this aspect is surprising and state: "The optimal values of the active-to-passive drag (λ) imply that the drag of an actively swimming animal is less than that for a passive animal. Although recent work on oscillating foils (Triantafyllou et al. 1993) lends support to this idea, there are a number of complications that make it difficult to reach a firm conclusion."

---

[4] Gough et al. (2019) Scaling of swimming performance in baleen whales | Journal of Experimental Biology | The Company of Biologists
Gough et al. (2021) Scaling of oscillatory kinematics and Froude efficiency in baleen whales | The Company of Biologists
[5] Hind and Giurney (1997) The metabolic cost of swimming in marine homeotherms | The Company of Biologists
