# Supplementary material for: Round-trip migration and energy budget of a breeding female humpback whale in the Northeast Atlantic
Source: PLoS One. 2022 May 27;17(5):e0268355. doi: 10.1371/journal.pone.0268355 (PMC9140263; doi:10.1371/journal.pone.0268355)
Supplement: Attachment — Submitted filename: Response_PLOS_final.pdf [file pone.0268355.s005.pdf]

Abstract (line 38): consider editing the sentence to say "…presumably in response to the calf's needs after its birth."

Implemented.

Methods: Sometimes there seems to be an extra space between a function name and the brackets.

Corrected.

There are some issues with the figure legends:
Lines 111 and 120 both are legends for 'Figure 1', and therefore Line 211 Fig 2 legend should in fact be Fig 3, and line 245 Fig 3 legend should in fact be Fig 4.
Line 111 for Fig 1: the legend states that "Tagging location is indicated by a red triangle and the putative location of birth is indicated by a red star." But these are in fact denoted by yellow/orange triangle and square. Furthermore, the legend text about the move persistence states that "lighter blue colors indicating directional travel, and darker blue colors indicating meandering movement." whereas the color legend ranges from yellow to dark blue.

Corrected.

Line 255: "…was only detected after the pair transited to Navidad Bank on May 1st (Fig. 2)". – I think this line should probably be referring to Fig 1 or Fig 3, as Fig 2 is pictures of the whales…?

Corrected.

Line 266-267: "We also present a theoretical cost at lower migration speeds (as reported in previous studies, 0.9 m s-1 and 1.1 m s-1) in S1 Table in S1 Supporting information." – I this line should be referring to Table S2?

Corrected.

There has likely been some issue in submitting Figure S2 as nothing is plotted in the image.

In our version of the pdf compiled submission material this figure is visible. We will check whether this problem consists in the following process.

Lines 376-378: "The fast migration speed and long distance resulted in a high energetic cost of this migration, compared to e.g., lactating females migrating 0.6 ms-1 slower and ~ half the distance from the Southern Ocean to Australia [7]." -- in [7] the migration direction was in fact from Australia/north of New Zealand towards Southern Ocean, not from the Southern Ocean to Australia.

Corrected.
